# A primary study: high-throughput sequencing analysis of amniotic fluid microbiota in 50 high-risk pregnant women during the second trimester

**DOI:** 10.3389/fcimb.2026.1748232

**Published:** 2026-04-22

**Authors:** Junhua Wu, Yanbo Lu, Zhouyong Zheng, Jishan Zheng, Qiang Fu

**Affiliations:** 1 Department of Pediatrics, The affiliated Women and Children’s Hospital of Ningbo University, Ningbo, Zhejiang, China; 2 Department of Pediatrics, Jingzhou Hospital Affiliated to Yangtze University, Jingzhou, Hubei, China

**Keywords:** amniocentesis, amniotic fluid, high-risk pregnant, microbiome, next generation sequencing, second trimester

## Abstract

**Objective:**

Whether bacteria exist in the pregnancy uterus without pathological infection has long been a controversial topic. Through this study, we aim to determine whether characteristic amniotic fluid microbiota exists in the uterus of high-risk pregnant women during the second trimester.

**Methods:**

This study recruited high-risk pregnant women in the second trimester from September 1, 2024, to August 30, 2025, and recorded their age and gestational age. Amniotic fluid samples were obtained through amniocentesis under strictly sterile conditions, and 16S rRNA high-throughput sequencing was performed on the samples. The inclusion criteria mainly consisted of: advanced maternal age, non-invasive prenatal test results indicating chromosomal abnormalities, abnormal fetal ultrasound findings, history of adverse pregnancy outcomes, and high-risk Down syndrome screening results.

**Results:**

This study included a total of 50 high-risk pregnant women who underwent amniocentesis. The results showed that bacteria were present in all amniotic fluid samples, primarily composed of *Actinobacteriota* and *Proteobacteria*. There was no difference in amniotic fluid microbiota diversity between pregnant women under 35 years old and those 35 years or older; however, the abundances of *Cutibacterium* and *Leifsonia* differed between the two groups. A slight difference in microbiota diversity was observed between women with gestational ages below 20 weeks and those at 20 weeks or above, but no significant difference was found in microbial composition between the two groups.

**Conclusion:**

There was bacterial DNA in amniotic fluid of high-risk pregnant women in the second trimester, with *Actinobacteriota* and *Proteobacteria* being the predominant phyla, showing limited correlation with maternal age or gestational age. This study provided evidence for the presence of microorganisms in amniotic fluid during pregnancy and might offer some preliminary data for future research related to eugenics and reproductive health.

## Introduction

The initial microbial colonization critically shapes early-life development, affecting immune, metabolic, and physiological functions with lasting health implications (Lim et al., 2016). Although pivotal, the origin and exact timing of the first microbial colonization in humans remain controversial (Banchi et al., 2023). For a long time, it has been widely believed that during a normal pregnancy, the growth and development of the fetus in the uterus occur in a sterile environment, unless pathological conditions arise (Romero et al., 2007). Leveraging advances in metagenomics and 16S rRNA gene amplicon sequencing, recent studies have challenged this notion by detecting bacterial DNA in placental tissue, amniotic fluid, and even developing fetuses (Collado et al., 2016; Seferovic et al., 2019; Collado and Segata, 2020).

The existence of intrauterine microbiota during normal pregnancy remains controversial (Collado et al., 2016; de Goffau et al., 2019). These studies have reported a wide range of findings: some detected no bacteria in amniotic fluid (Liu et al., 2020; Jung et al., 2021), others found bacteria present in all amniotic fluid samples (Zhu et al., 2018; Wang et al., 2021), while several reported a combination of both positive and negative samples (Stinson et al., 2019; Campisciano et al., 2021). Stinson et al (Stinson et al., 2020). found that amniotic fluid samples contained bacterial DNA profiles of low abundance and diversity, which showed no association with spontaneous preterm birth. Subsequently, Wang et al (Wang et al., 2021). showed distinct microbial compositions in amniotic fluid between normal pregnancies and advanced maternal age pregnancies, with all delivered neonates being healthy and devoid of allergic manifestations. These findings challenged the prevailing notion that the presence of microbes in amniotic fluid was universally linked to adverse pregnancy outcomes. Through analysis of shared OTUs between meconium microbiota and various maternal microbiota samples, He et al (He et al., 2020). discovered that meconium microbiota shared more features with amniotic fluid microbiota than with maternal fecal and vaginal microbiota, with amniotic fluid microbiota exerting the greatest influence on meconium microbiota. Staude et al (Staude et al., 2023). demonstrated that preterm infants exhibited distinct amniotic fluid microbiota profiles compared to term infants prior to birth. Furthermore, preterm neonates with varying severity of bronchopulmonary dysplasia at birth showed differential amniotic fluid microbial characteristics. These findings underscore the significant influence of prenatal intrauterine microbiota on the pathogenesis of bronchopulmonary dysplasia. Therefore, information regarding the presence and significance of microorganisms in amniotic fluid is of paramount importance.

Existing studies on the microbiome of high-risk pregnant women have primarily focused on the vaginal microbiota, as vaginal dysbiosis during pregnancy appears to be linked to preterm birth or miscarriage (Chang et al., 2020; Huang et al., 2022; Schuster et al., 2024). The upward migration of vaginal microbiota into the uterus may be one potential source of the amniotic fluid microbiome (Dera et al., 2024). Investigating the amniotic fluid microbiome could play a crucial role in advancing research on maternal microbial ecology during pregnancy. Current researches predominantly characterized intrauterine microbiota using samples collected during delivery, which might be contaminated by vaginal or cutaneous microorganisms during the birthing process (Zhu et al., 2018; Stinson et al., 2019; Campisciano et al., 2021). In contrast, amniotic fluid samples obtained via mid-trimester amniocentesis represent an ideal specimen type for accurately reflecting the authentic microbial composition within the uterine environment during pregnancy (Zemet et al., 2025). Therefore, this preliminary study collected amniotic fluid samples in high-risk pregnant women during the second trimester through amniocentesis and analyzed bacterial DNA profiles in mid pregnancy human amniotic fluid through V4 region 16S rRNA gene sequencing.

## 2 Methods

### 2.1 Participants

This is an observational cohort study conducted from September 1, 2024 to August 30, 2025 at The Affiliated Women and Children’s Hospital of Ningbo University in Zhejiang Province, China. We enrolled pregnant women who received amniocentesis at the hospital during this period and documented their age and gestational age. The primary indications for amniocentesis include: advanced maternal age, non-invasive prenatal testing results indicative of chromosomal abnormalities, abnormal fetal ultrasound findings, history of adverse pregnancy outcomes, and high-risk Down syndrome screening results. Exclusion criteria comprised: Individuals with acute genital tract inflammation; History of psychiatric disorders; Severe cardiovascular, digestive, or other systemic diseases; Family history of genetic diseases. Each participant provided written informed consent. The study was approved by the Ethics Committee of Ningbo Women and Children’s Hospital and was conducted in accordance with the Declaration of Helsinki.

Since 20 weeks of gestation is a common clinical cutoff for undergoing amniocentesis for prenatal diagnosis, and 35 years of age is the standard threshold for defining ‘advanced maternal age,’ in the subsequent analysis we compared the study subjects based on whether they were before or after 20 weeks of gestation and before or after 35 years of age.

### 2.2 Sampling

The puncture site was thoroughly disinfected. Approximately 4 mL of amniotic fluid was aseptically collected into sterile specialized tubes during the procedure and immediately preserved in an ultra-low temperature freezer (-80 °C) for subsequent microbiome analysis.

### 2.3 DNA extraction and 16S rRNA pyrosequencing

In this study, we amplified and sequenced the bacterial V4 region. Microbial DNA was extracted from amniotic fluid using the QIAamp DNA Microbiome Kit (Qiagen) following the manufacturer’s instructions. The V4 region of qualified DNA products was amplified using primers (515F: GTGCCAGCMGCCGCGGTAA, 806R: GGACTACHVGGGTWTCTAAT). PCR amplification was performed under the following conditions: 95 °C for 5 min; 35 cycles of 95 °C for 30 s, 56 °C for 30 s, 72 °C for 30 s; and a final extension at 72 °C for 7 min. Quantification and purification of PCR products were performed using the Quant-iT™ PicoGreen^®^ dsDNA Assay Kit and QIAquick PCR Purification Kit (Qiagen), respectively. The recovered products were amplified in a second round with the introduction of Illumina bridge PCR-compatible primers. Amplification products were quantified by fluorometry (PicoGreen) and analyzed using an Agilent 2200 Bioanalyzer. After qualification, sequencing was performed on the Illumina MiSeq platform. All experimental procedures were conducted in strict accordance with the manufacturers’ protocols. In order to reduce background DNA pollution, we took the following measures: including (1) filtering all reagents with 0.2 µ M filter membrane; (2) Ung enzyme was added into the PCR premix to prevent carryover contamination; (3) No template control was set for each batch, and the CT value was greater than 35.

### 2.4 Bioinformatics analysis

Raw data obtained from the Illumina platform underwent quality control using fastp software to trim low-quality bases and adapter sequences. The DADA2 plugin in QIIME2 (version 2022.8) was employed for sequence denoising to generate amplicon sequence variants (ASVs) and the feature table. Subsequently, taxonomic annotation of ASV sequences was performed using the RDP classifier (version 2.13) with a confidence threshold of 0.7. The Silva 138.1 database served as the reference for bacterial taxonomy. Alpha diversity indices were calculated using mothur v1.43.0. Principal Coordinates Analysis (PCoA) was conducted with the vegan package v2.5–6 in R.

### 2.5 Statistical analyses

Alpha diversity differences were assessed by Student’s t-test. Beta diversity was calculated using the Bray-Curtis distance, and PCoA was performed based on the resulting matrix. Bacterial abundance variations were analyzed using Linear Discriminant Analysis Effect Size (LEfSe), combining Kruskal-Wallis tests for significance detection and Linear Discriminant Analysis (LDA) for effect size visualization. For all results, P < 0.05 was considered statistically significant.

## 3 Result

### 3.1 Participants’ characteristics

The study enrolled 50 high-risk pregnant women, including 32 with gestational age <20 weeks and 18 with ≥20 weeks. Seventeen women were ≥35 years old, and 33 were <35 years old. Among the 50, 17 were of advanced maternal age, 13 had abnormal non-invasive DNA results, 12 showed abnormal ultrasound findings, 11 had a history of adverse pregnancy outcomes, 8 were at high risk for Down syndrome screening, and 2 had other abnormalities—one with bilateral ear malformations and another where both the pregnant woman and her father exhibited significant exophthalmos (**Table 1**).

**Table 1:** Participant’s characteristics.

Information	
Age (years)	32 (28, 36)
Gestational weeks (weeks)	17 (15, 21)
High risk factors (numbers)	The number of cases
advanced age	17
Non-invasive DNA abnormality	13
Abnormal ultrasound findings	12
Adverse pregnancy history	11
High risk in Down screening	8
Others	2

Age and gestational age are represented by median and quartiles, High risk factors are the number of cases of pregnant women.

### 3.2 The overall microbial characteristics of amniotic fluid in 50 pregnant women

**Figure 1:**
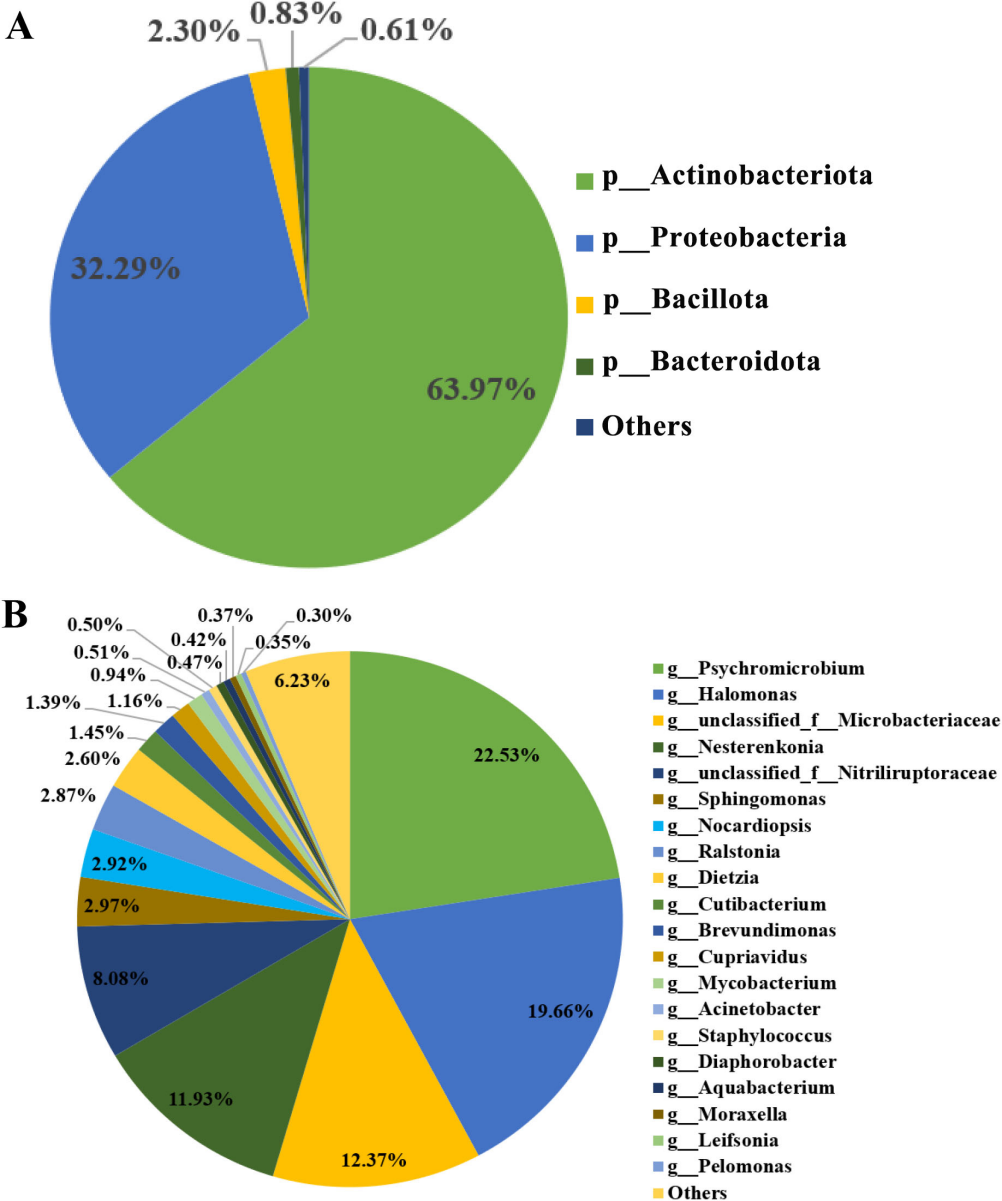
Pie chart of relative abundance composition of amniotic fluid microbiota in 50 pregnant women **(A)** Composition of main microbiota at phylum level; **(B)** Composition of main microbiota at genus level.

A total of 5,984,875 filtered sequences were obtained from the sequencing of the 50 samples, with a median of 122,390. A total of 970 ASVs were detected. The bacteria detected included 22 phyla and 476 genera. The most abundant phyla were *Actinobacteriota* (63.97% ± 6.49%), *Proteobacteria* (32.29% ± 6.43%), *Bacillota* (2.3% ± 1.85%) and *Bacteroidota* (0.83% ± 0.51%). The relative abundance of these four phyla accounted for 99.39% ± 1.23% (**Figure 1A**).

At the genus level, the most abundant genera were *Psychromicrobium* (22.53% ± 8.40%), *Halomonas* (19.66% ± 6.44%), *unclassified_Microbacteriaceae* (12.37% ± 4.04%), *Nesterenkonia* (11.93% ± 4.08%), *unclassified_Nitriliruptoraceae* (8.08% ± 2.35%), *Sphingomonas* (2.97% ± 1.13%), *Nocardiopsis* (2.92% ± 1.19%), *Ralstonia* (2.87% ± 1.25%), *Dietzia* (2.60% ± 0.94%), *Cutibacterium* (1.45% ± 1.93%), *Brevundimonas* (1.39% ± 1.37%), *Cupriavidus* (1.16% ± 0.38%), *Mycobacterium* (0.94% ± 0.38%), *Acinetobacter* (0.51% ± 0.55%), *Staphylococcus* (0.50% ± 0.53%), *Diaphorobacter* (0.47% ± 0.46%), *Aquabacterium* (0.42% ± 0.27%), *Moraxella* (0.37% ± 0.44%), *Leifsonia* (0.35% ± 0.25%) and *Pelomonas* (0.30% ± 0.23%). The relative abundance of these twenty genera accounted for 93.77% ± 2.75% (**Figure 1B**).

### 3.3 Comparative analysis of amniotic fluid microbiota characteristics in pregnant women under vs. over 35 years old

**Figure 2:**
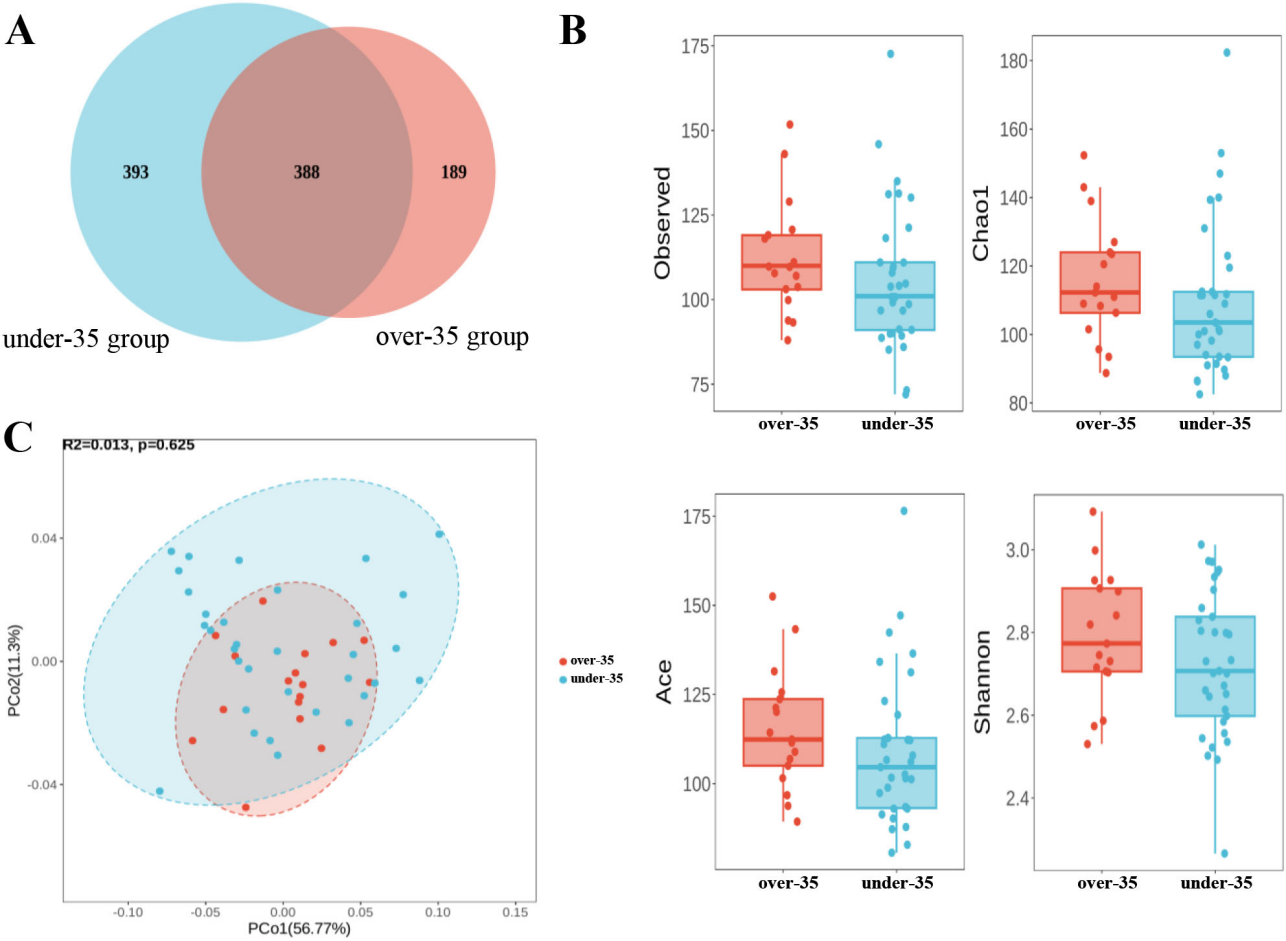
Analysis of amniotic fluid microbiota diversity in pregnant women between aged ≥35 years and <35 years **(A)** Venn diagram of ASV composition; **(B)** Box plot of alpha diversity comparison; **(C)** Beta diversity comparison by PCoA.

Among the 50 pregnant women, 17 were assigned to the ≥35-year-old group, and 33 were assigned to the <35-year-old group. In terms of ASV composition, a total of 388 ASVs were shared between the two groups, with 189 ASVs unique to the over-35 group and 393 ASVs unique to the under-35 group (**Figure 2A**). We analyzed the alpha diversity indices of the amniotic fluid microbiota in two groups of pregnant women, including Observed, Chao1, ACE and Shannon. The results showed no statistically significant differences in alpha diversity indices between the two groups, indicating that there were no notable differences in species diversity and richness of the amniotic fluid microbiota between the two groups of pregnant women (**Figure 2B**, **Table 2**). Beta diversity analysis using PCoA revealed that samples from both groups fell within the 95% confidence interval, with no outliers observed (**Figure 2C**). The microbial community composition exhibited group-specific clustering patterns between the two groups, indicating some differences in the composition of amniotic fluid microbiota between the over-35 group and the under-35 group. The first principal component explained 56.77% of the variation, while the second principal component accounted for 11.3% of the differences.

**Table 2:** Comparison of alpha diversity in amniotic fluid microbiota between over-35 group and under-35 group.

Alpha-diversity index	Over-35 group	Under-35 group	p value
Observed	112.35 ± 16.98	105.97 ± 21.05	0.285
Chao1	115.86 ± 17.60	109.69 ± 22.30	0.327
ACE	115.20 ± 16.91	108.86 ± 20.86	0.284
Shannon	2.79 ± 0.15	2.72 ± 0.17	0.166

**Figure 3:**
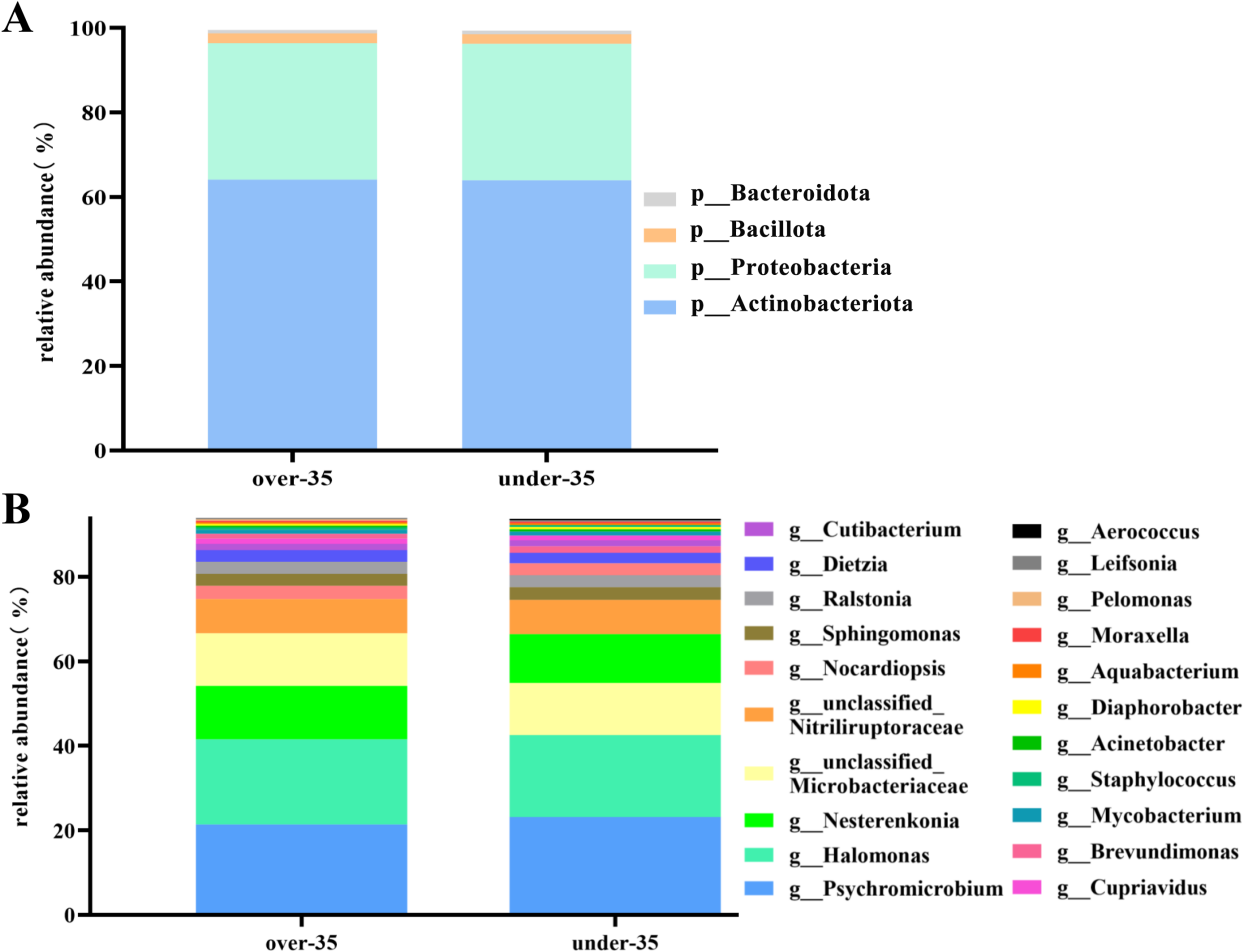
The composition of amniotic fluid microbiota in pregnant women aged ≥35 years and <35 years **(A)** Microbiota composition at the phylum level; **(B)** Microbiota composition at the genus level.

We analyzed the composition of amniotic fluid microbiota at the phylum and genus levels in two groups of pregnant women. At the phylum level (**Figure 3A**), the top four phyla in the over-35 group were *Actinobacteriota* (64.06%), *Proteobacteria* (32.32%), *Bacillota* (2.35%), and *Bacteroidota* (0.78%), collectively accounting for 99.51% of the relative abundance. In the under-35 group, the top four phyla were the same as those in the over-35 group, with relative abundances of 63.93%, 32.28%, 2.27%, and 0.85%, respectively, totaling 99.33%.

At the genus level (**Figure 3B**), the top twenty genera in the over-35 group accounted for 93.95% of the total. The top twenty genera in the over-35 group were *Psychromicrobium* (21.34%), *Halomonas* (20.21%), *Nesterenkonia* (12.62%), *unclassified_Microbacteriaceae* (12.45%), *unclassified_Nitriliruptoraceae* (8.10%), *Nocardiopsis* (3.15%), *Sphingomonas* (2.85%), *Ralstonia* (2.83%), *Dietzia* (2.79%), *Cutibacterium* (1.46%), *Cupriavidus* (1.22%), *Brevundimonas* (1.16%), *Mycobacterium* (0.97%), *Staphylococcus* (0.55%), *Acinetobacter* (0.42%), *Diaphorobacter* (0.40%), *Aquabacterium* (0.39%), *Moraxella* (0.37%), *Pelomonas* (0.36%) and *Leifsonia* (0.33%).

The top twenty genera in the under-35 group accounted for 93.71% of the total. The top twenty genera in the under-35 group were *Psychromicrobium* (23.15%), *Halomonas* (19.37%), *unclassified_Microbacteriaceae* (12.33%), *Nesterenkonia* (11.57%), *unclassified_Nitriliruptoraceae* (8.06%), *Sphingomonas* (3.03%), *Ralstonia* (2.89%), *Nocardiopsis* (2.80%), *Dietzia* (2.49%), *Brevundimonas* (1.51%), *Cutibacterium* (1.44%), *Cupriavidus* (1.13%), *Mycobacterium* (0.92%), *Acinetobacter* (0.56%), *Diaphorobacter* (0.51%), *Staphylococcus* (0.48%), *Aquabacterium* (0.43%), *Moraxella* (0.37%), *Leifsonia* (0.36%) and *Aerococcus* (0.31%).

**Figure 4:**
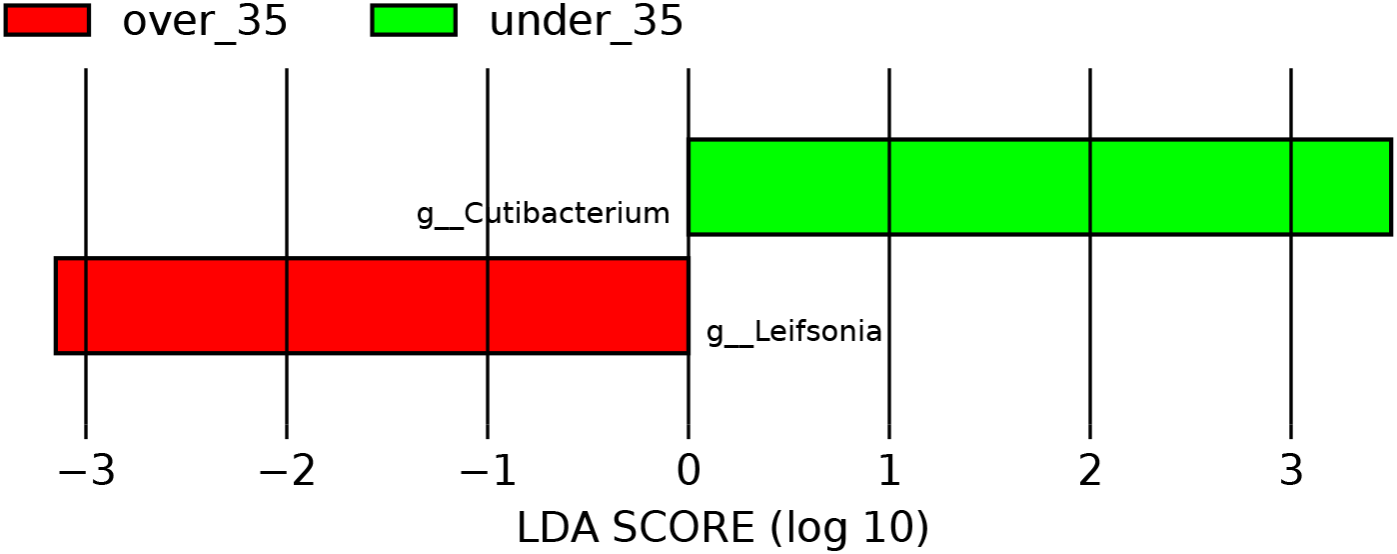
Histogram of LDA effect values for marker species.

We performed differential analysis on the top 4 phyla and top 20 genera representing the overall microbial composition. The results showed that the relative abundances of *Cutibacterium* (p=0.0059) was significantly higher in the amniotic fluid of the pregnant women in the under-35 group compared to those in the over-35 group. Conversely, the relative abundance of *Leifsonia* (p<0.0001) was higher in the over-35 group (**Figure 4**).

### 3.4 Comparative analysis of amniotic fluid microbiome characteristics between pregnant women with gestational age above and below 20 weeks

**Figure 5:**
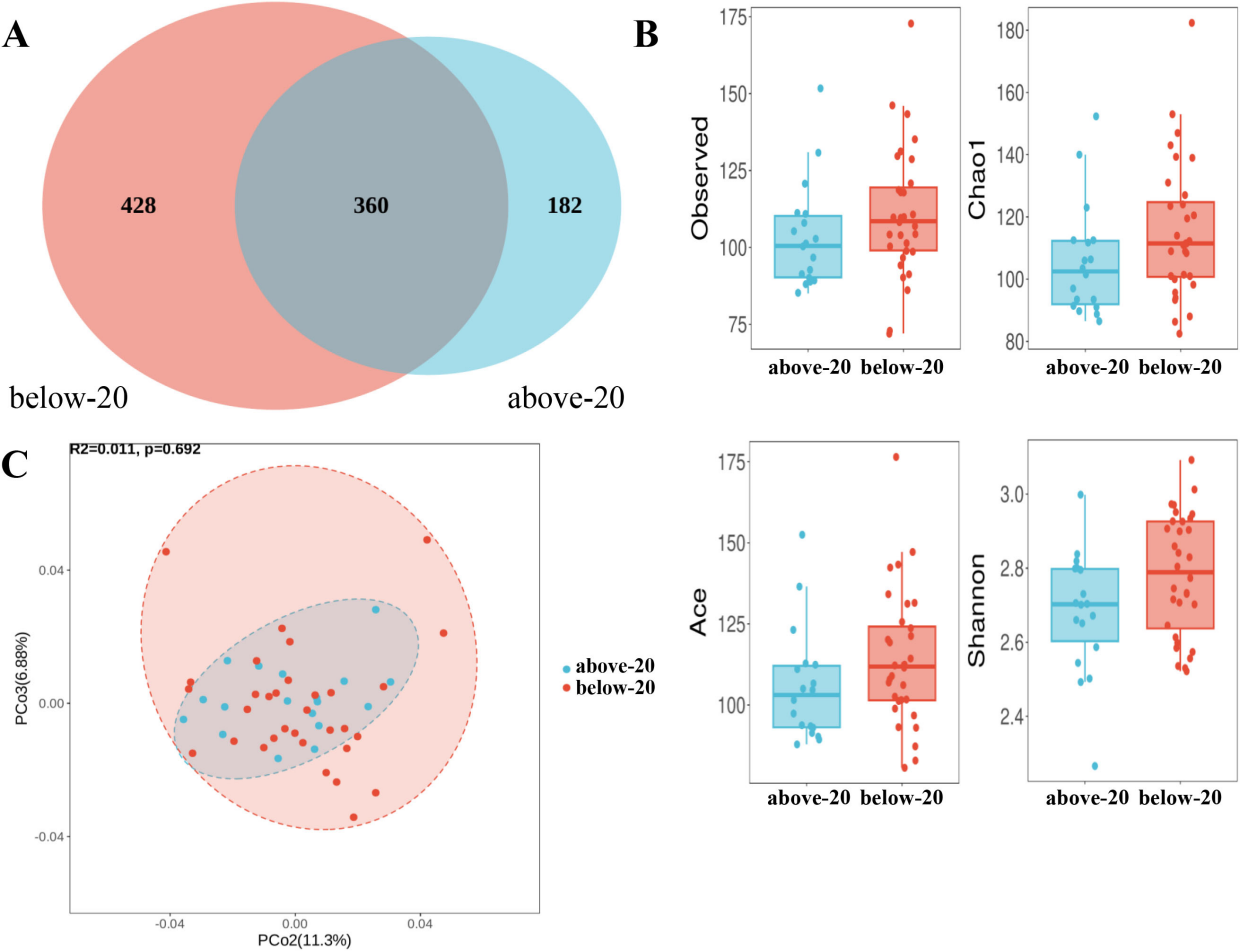
Analysis of amniotic fluid microbiota diversity in pregnant women between gestational age ≥20 weeks and <20 weeks **(A)** Venn diagram of ASV composition; **(B)** Box plot of alpha diversity comparison; **(C)** Beta diversity comparison by PCoA.

Among the 50 pregnant women, 32 had a gestational age of less than 20 weeks, while 18 were beyond 20 weeks. In terms of ASV composition, a total of 360 ASVs were shared between the two groups, with 182 ASVs unique to the above-20 group and 428 ASVs unique to the below-20 group (**Figure 5A**). The results of the alpha diversity indices showed that the Shannon index was significantly higher in the group with gestational age below 20 weeks compared to the group above 20 weeks (**Figure 5B**, **Table 3**). Beta diversity analysis showed that the microbial community composition exhibited group-specific clustering patterns between the two groups, indicating some differences in the composition of amniotic fluid microbiota between the above-20 group and the below-20 group. The first principal component explained 11.3% of the variation, while the second principal component accounted for 6.8% of the differences (**Figure 5C**).

**Table 3:** Comparison of alpha diversity in amniotic fluid microbiota between above-20 group and below-20 group.

Alpha-diversity index	Above-20 group	Below-20 group	p value
Observed	103.61 ± 17.26	110.69 ± 20.96	0.229
Chao1	105.60 ± 18.00	115.27 ± 21.78	0.116
ACE	105.70 ± 17.39	114.01 ± 20.49	0.154
Shannon	2.68 ± 0.16	2.78 ± 0.16	0.041

**Figure 6:**
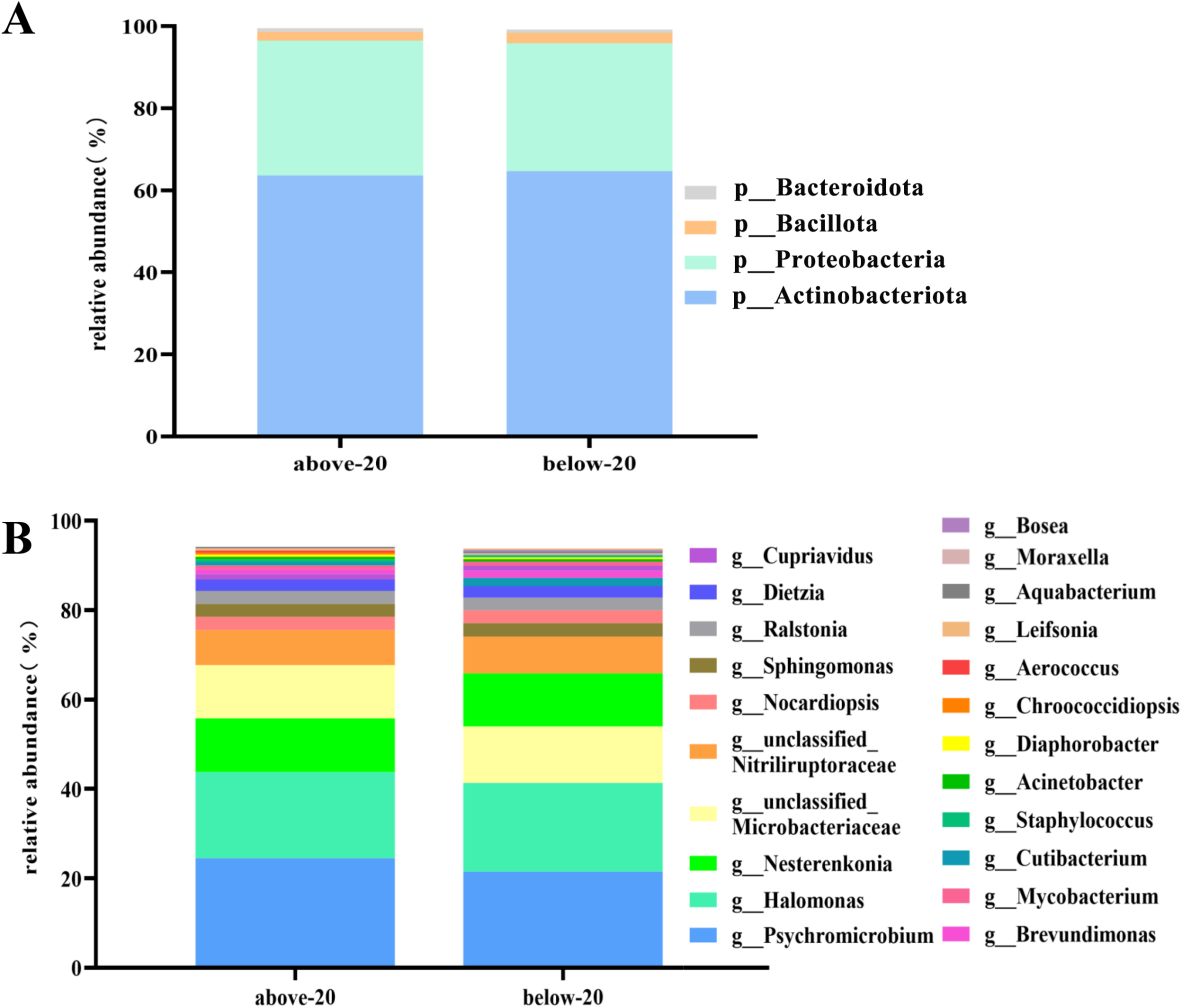
The composition of amniotic fluid microbiota in pregnant women with gestational age <20 weeks and ≥20 weeks. **(A)** Microbiota composition at the phylum level; **(B)** Microbiota composition at the genus level.

We characterized the major bacterial communities in the amniotic fluid from the two pregnancy groups. At the phylum level (**Figure 6A**), the top four phyla in the above-20 group were *Actinobacteriota* (64.65%), *Proteobacteria* (31.22%), *Bacillota* (2.53%), and *Bacteroidota* (0.79%), collectively accounting for 99.19% of the relative abundance. In the below-20 group, the top four phyla were the same as those in the above-20 group, with relative abundances of 63.59%, 32.89%, 2.17%, and 0.85%, respectively, totaling 99.50%.

At the genus level (**Figure 6B**), the top twenty genera in the above-20 group accounted for 94.15% of the total. The top twenty genera in the above-20 group were *Psychromicrobium* (24.47%), *Halomonas* (19.27%), *Nesterenkonia* (12.05%), *unclassified_Microbacteriaceae* (11.90%), *unclassified_Nitriliruptoraceae* (7.83%), *Nocardiopsis* (2.98%), *Sphingomonas* (2.92%), *Ralstonia* (2.91%), *Dietzia* (2.53%), *Cupriavidus* (1.20%), *Brevundimonas* (1.06%), *Mycobacterium* (0.86%), *Cutibacterium* (0.76%), *Staphylococcus* (0.62%), *Acinetobacter* (0.57%), *Diaphorobacter* (0.47%), *Chroococcidiopsis* (0.46%), *Aerococcus* (0.45%), *Leifsonia* (0.43%) and *Aquabacterium* (0.40%).

The top twenty genera in the below-20 group accounted for 93.76% of the total. The top twenty genera in the below-20 group were *Psychromicrobium* (21.44%), *Halomonas* (19.87%), *unclassified_Microbacteriaceae* (12.64%), *Nesterenkonia* (11.86%), *unclassified_Nitriliruptoracea*e (8.22%), *Sphingomonas* (2.99%), *Nocardiopsis* (2.88%), *Ralstonia* (2.84%), *Dietzia* (2.63%), *Cutibacterium* (1.83%), *Brevundimonas* (1.57%), *Cupriavidus* (1.13%), *Mycobacterium* (0.98%), *Acinetobacter* (0.48%), *Diaphorobacter* (0.47%), *Staphylococcus* (0.44%), *Moraxella* (0.44%), *Aquabacterium* (0.43%), *Bosea* (0.32%) and *Leifsonia* (0.30%).

The differential analysis results of the top 4 phyla and top 20 genera in relative abundance showed no statistically significant differences between the two groups.

## Discussion

In this study, amniotic fluid samples were collected via amniocentesis and analyzed using 16S rRNA sequencing to characterize the mid-trimester amniotic fluid microbiota in 50 pregnant women with high-risk factors. We compared the microbial profiles between women under 35 years and those 35 years or older, as well as between pregnancies at ≤20 weeks’ gestation and those >20 weeks. Our findings provided evidence for the presence of microorganisms in amniotic fluid during gestation.

The high-risk factors among pregnant women undergoing amniocentesis in this study primarily included advanced maternal age, abnormal non-invasive DNA results, abnormal ultrasound findings, history of adverse pregnancy outcomes, and high-risk screening for Down syndrome. The study by Huang et al. found distinct differences in the vaginal and gut microbiota of women with advanced maternal age compared to women with non-advanced maternal age and non-pregnant individuals (Huang et al., 2022). Specifically, *Bifidobacterium* was significantly enriched in the vaginal microbiota, while *Prevotella bivia* showed notable abundance in the gut microbiome. The studies by Zhang (Zhang et al., 2025) and Chen (Chen et al., 2022) et al. have consistently shown that the amniotic fluid metabolome of pregnant women carrying fetuses with Down syndrome is significantly altered compared to that of the control group. These studies suggest potential associations between microbial composition/metabolism and high-risk pregnancy factors or complication risks, warranting further investigation.

Our findings showed the following mid-trimester amniotic fluid microbiota composition in high-risk pregnancies: At the phylum level, the microbiota was predominantly composed of *Actinobacteriota* and *Proteobacteria*. At the genus level, the most abundant taxa were *Psychromicrobium*, *Halomonas*, *unclassified*_*Microbacteriaceae*, *Nesterenkonia*, and *unclassified*_*Nitriliruptoraceae*. The phyla *Actinobacteriota* and *Proteobacteria* were detected in maternal vaginal samples and were found to be the dominant bacterial taxa in neonatal oral specimens (Xu et al., 2022; Silvano et al., 2023). Interestingly, while *Bacillota* has been reported as the dominant bacterial phylum in previous studies—whether in maternal vaginal samples (Silvano et al., 2023), neonatal oral cavities (Xu et al., 2022), or meconium (Turunen et al., 2024)—it accounted for only 2.3% of the microbiota in this study. This finding may suggested that direct perinatal factors exerted a greater influence on neonatal microbial colonization than prenatal factors. One study found that before pregnancy, the female vaginal microbiota was mainly composed of *Bacillota*, followed by *Actinobacteria* and *Proteobacteria*. However, after childbirth, *Bacillota* in vaginal microorganisms decreased, and the abundance of *Actinobacteria* and *Proteobacteria* increased (Hu et al., 2023). A cohort study showed that the meconium microbiota was composed of *Bacillota*, *Proteobacteria* and *Actinobacteria* and pointed out that perinatal factors had more influence on the composition of meconium microbiota than prenatal factors (Turunen et al., 2024). These conclusions supported our inference.

There was no difference in amniotic fluid microbiota diversity between pregnant women under 35 years old and those 35 years or older; however, the abundances of *Cutibacterium* and *Leifsonia* differed between the two groups. In this study, the alpha diversity indices of the amniotic fluid microbiota were relatively low, significantly lower than those of neonatal gut microbiota (Chen et al., 2023). This finding aligned with our general understanding, as the bacterial load in amniotic fluid was substantially lower than that in the neonatal gastrointestinal tract. *Cutibacterium* was a commensal skin bacterium and maintained skin homeostasis, while dysbiosis of its abundance may induced cutaneous inflammation (Boyanova, 2023). A study combining amniotic fluid culture and sequencing revealed that cultivable microorganisms were detected in 33.1% of samples, with *Cutibacterium* being common, suggesting that viable bacteria and/or DNA can transiently enter the microbial balance of the gestational environment (González-Rovira et al., 2026). *Leifsonia* is a Gram-positive rod-shaped bacterium that has been reported to be associated with central venous catheter-related infections (Al-Sardi et al., 2021). Campisciano et al. detected bacterial DNA in chorionic villi and amniotic fluid during the first and second trimesters, with the identified bacterial DNA belonging to taxa commonly found in the vaginal and skin microbiomes (Campisciano et al., 2021). The mechanisms by which these two bacterial genera entered the amniotic fluid and their potential roles in this environment required further investigation.

Our results showed minor differences in microbiota diversity between women at <20 weeks and ≥20 weeks of gestation, but microbial composition did not differ significantly between the groups. The study by Stinson et al. found that the bacterial DNA profile in amniotic fluid is characterized by low abundance and low diversity, and there was no significant difference in the bacterial DNA profile of mid-trimester amniotic fluid between spontaneous preterm birth and full-term delivery (Stinson et al., 2020). Sharlandjieva et al. found that bacterial abundance in the placenta showed a weak positive correlation with gestational age, but this was not statistically significant, and bacterial diversity did not change significantly with increasing gestational age (Sharlandjieva et al., 2023). The bacterial diversity and composition in amniotic fluid may be independent of gestational age, though more in-depth studies are required to confirm this.

This observational study employed high-throughput sequencing of mid-trimester amniotic fluid from high-risk pregnancies, providing preliminary insights into the characteristics of the amniotic fluid microbiota. However, this initial investigation had several limitations, including a relatively small sample size and the lack of correlation between these amniotic fluid samples and pregnancy outcomes. In addition, the bacterial DNA obtained by sequencing could not determine the bacterial activity. In future studies, we plan to continue collecting amniocentesis specimens and follow up with participants, and further verify them by combining amniotic fluid culture and *in situ* staining. When feasible, we will also collect samples (such as skin swabs, oral swabs, and meconium) from neonates delivered at our hospital to enable a more comprehensive exploration of maternal-fetal microbial ecology during pregnancy and the perinatal period.

## Conclusion

There was bacterial DNA in amniotic fluid of high-risk pregnant women in the second trimester, with *Actinobacteriota* and *Proteobacteria* being the predominant phyla, showing limited correlation with maternal age or gestational age. This study provided evidence for the presence of microorganisms in amniotic fluid during pregnancy and might offer some preliminary data for future research related to eugenics and reproductive health.

## Data Availability

The datasets presented in this study can be found in online repositories. The names of the repository/repositories and accession number(s) can be found below: https://www.ncbi.nlm.nih.gov/, SUB15754158.

## References

[B1] Al-Sardi M. Radwan H. Itbaileh A. B. AlMusa Z. (2021). Leifsonia species bacteremia in a hemodialysis patient: a difficult-to-identify organism. Cureus 13, e17994. doi: 10.7759/cureus.17994. PMID: 34540513 PMC8442807

[B2] Banchi P. Colitti B. Opsomer G. Rota A. Van Soom A. (2023). The dogma of the sterile uterus revisited: does microbial seeding occur during fetal life in humans and animals? Reproduction 167, e230078. doi: 10.1530/rep-23-0078. PMID: 37903182 PMC10762539

[B3] Boyanova L. (2023). Cutibacterium acnes (formerly Propionibacterium acnes): friend or foe. Future Microbiol. 18, 235–244. doi: 10.2217/fmb-2022-0191, PMID: 37042433

[B4] Campisciano G. Quadrifoglio M. Comar M. De Seta F. Zanotta N. Ottaviani C. . (2021). Evidence of bacterial DNA presence in chorionic villi and amniotic fluid in the first and second trimester of pregnancy. Future Microbiol. 16, 801–810. doi: 10.2217/fmb-2020-0243. PMID: 34223788

[B5] Chang D. H. Shin J. Rhee M. S. Park K. R. Cho B. K. Lee S. K. . (2020). Vaginal microbiota profiles of native Korean women and associations with high-risk pregnancy. J. Microbiol. Biotechnol. 30, 248–258. doi: 10.4014/jmb.1908.08016. PMID: 31838792 PMC9728229

[B6] Chen X. Hu L. Su J. Liu X. Luo X. Pei Y. . (2022). Amniotic fluid and urine metabolomic alterations associated with pregnant women with Down syndrome fetuses. J. Mater Fetal Neonatal Med. 35, 7882–7889. doi: 10.1080/14767058.2021.1937990. PMID: 34130603

[B7] Chen Y. Lu Y. Wang T. Wu J. Yu B. (2023). Changes in gut microbiota at 1–60 days in 92 preterm infants in a neonatal intensive care unit using 16S rRNA gene sequencing. Med. Sci. Monit. 29, e941560. doi: 10.12659/msm.941560. PMID: 38018034 PMC10699091

[B8] Collado M. C. Rautava S. Aakko J. Isolauri E. Salminen S. (2016). Human gut colonisation may be initiated in utero by distinct microbial communities in the placenta and amniotic fluid. Sci. Rep. 6, 23129. doi: 10.1038/srep23129. PMID: 27001291 PMC4802384

[B9] Collado M. C. Segata N. (2020). Initial exploration of in utero microbial colonization. Nat. Med. 26, 469–470. doi: 10.1038/s41591-020-0836-1. PMID: 32231297

[B10] de Goffau M. C. Lager S. Sovio U. Gaccioli F. Cook E. Peacock S. J. . (2019). Human placenta has no microbiome but can contain potential pathogens. Nature 572, 329–334. doi: 10.1038/s41586-019-1451-5. PMID: 31367035 PMC6697540

[B11] Dera N. Żeber-Lubecka N. Ciebiera M. KosiŃska-KaczyŃska K. Szymusik I. Massalska D. . (2024). Intrauterine shaping of fetal microbiota. J. Clin. Med. 13, 5331. doi: 10.3390/jcm13175331. PMID: 39274545 PMC11396688

[B12] González-Rovira M. Sainz-Bueno J. A. García-Díaz L. Martínez-Pancorbo C. Sánchez J. Gutiérrez G. . (2026). Unveiling balanced prenatal microbial colonization in amniotic fluid through an integrated culture and sequencing approach. J. Transl. Med. 24, 273. doi: 10.1186/s12967-025-07601-0. PMID: 41514270 PMC12918369

[B13] He Q. Kwok L. Y. Xi X. Zhong Z. Ma T. Xu H. . (2020). The meconium microbiota shares more features with the amniotic fluid microbiota than the maternal fecal and vaginal microbiota. Gut Microbes 12, 1794266. doi: 10.1080/19490976.2020.1794266. PMID: 32744162 PMC7524391

[B14] Hu F. Sun X. Su Y. Huang M. (2023). The dynamic changes in the composition and diversity of vaginal microbiota in women of different pregnancy periods. Microorganisms 11, 2686. doi: 10.3390/microorganisms11112686. PMID: 38004698 PMC10673304

[B15] Huang Y. Li D. Cai W. Zhu H. Shane M. I. Liao C. . (2022). Distribution of vaginal and gut microbiome in advanced maternal age. Front. Cell. Infect. Microbiol. 12. doi: 10.3389/fcimb.2022.819802. PMID: 35694547 PMC9186158

[B16] Jung E. Romero R. Yoon B. H. Theis K. R. Gudicha D. W. Tarca A. L. . (2021). Bacteria in the amniotic fluid without inflammation: early colonization vs. contamination. J. Perinat Med. 49, 1103–1121. doi: 10.1515/jpm-2021-0191. PMID: 34229367 PMC8570988

[B17] Lim E. S. Wang D. Holtz L. R. (2016). The bacterial microbiome and virome milestones of infant development. Trends Microbiol. 24, 801–810. doi: 10.1016/j.tim.2016.06.001. PMID: 27353648

[B18] Liu Y. Li X. Zhu B. Zhao H. Ai Q. Tong Y. . (2020). Midtrimester amniotic fluid from healthy pregnancies has no microorganisms using multiple methods of microbiologic inquiry. Am. J. Obstet Gynecol 223, 248.e1–248.e21. doi: 10.1016/j.ajog.2020.01.056. PMID: 32017922

[B19] Romero R. Gotsch F. Pineles B. Kusanovic J. P. (2007). Inflammation in pregnancy: its roles in reproductive physiology, obstetrical complications, and fetal injury. Nutr. Rev. 65, S194–S202. doi: 10.1111/j.1753-4887.2007.tb00362.x. PMID: 18240548

[B20] Schuster H. J. Bos A. M. Himschoot L. van Eekelen R. Matamoros S. P. F. de Boer M. A. . (2024). Vaginal microbiota and spontaneous preterm birth in pregnant women at high risk of recurrence. Heliyon 10, e30685. doi: 10.1016/j.heliyon.2024.e30685. PMID: 38803950 PMC11128838

[B21] Seferovic M. D. Pace R. M. Carroll M. Belfort B. Major A. M. Chu D. M. . (2019). Visualization of microbes by 16S in situ hybridization in term and preterm placentas without intraamniotic infection. Am. J. Obstet Gynecol 221, 146.e1–146.e23. doi: 10.1016/j.ajog.2019.04.036. PMID: 31055031 PMC10357491

[B22] Sharlandjieva V. Beristain A. G. Terry J. (2023). Assessment of the human placental microbiome in early pregnancy. Front. Med. (Laus) 10. doi: 10.3389/fmed.2023.1096262. PMID: 36744135 PMC9892641

[B23] Silvano A. Meriggi N. Renzi S. Seravalli V. Torcia M. G. Cavalieri D. (2023). Vaginal microbiome in pregnant women with and without short cervix. Nutrients 15, 2173. doi: 10.3390/nu15092173. PMID: 37432374 PMC10180705

[B24] Staude B. Gschwendtner S. Frodermann T. Oehmke F. Kohl T. Kublik S. . (2023). Microbial signatures in amniotic fluid at preterm birth and association with bronchopulmonary dysplasia. Respir. Res. 24, 248. doi: 10.1186/s12931-023-02560-w. PMID: 37845700 PMC10577941

[B25] Stinson L. F. Boyce M. C. Payne M. S. Keelan J. A. (2019). The not-so-sterile womb: evidence that the human fetus is exposed to bacteria prior to birth. Front. Microbiol. 10. doi: 10.3389/fmicb.2019.01124. PMID: 31231319 PMC6558212

[B26] Stinson L. Hallingström M. Barman M. Viklund F. Keelan J. Kacerovsky M. . (2020). Comparison of bacterial DNA profiles in mid-trimester amniotic fluid samples from preterm and term deliveries. Front. Microbiol. 11. doi: 10.3389/fmicb.2020.00415. PMID: 32265868 PMC7107015

[B27] Turunen J. Tejesvi M. V. Paalanne N. Pokka T. Amatya S. B. Mishra S. . (2024). Investigating prenatal and perinatal factors on meconium microbiota: a systematic review and cohort study. Pediatr. Res. 95, 135–145. doi: 10.1038/s41390-023-02783-z. PMID: 37591927 PMC10798900

[B28] Wang Y. Luo C. Cheng Y. Li L. Liang D. Hu P. . (2021). Analysis of microbial differences in amniotic fluid between advanced and normal age pregnant women. J. Transl. Med. 19, 320. doi: 10.1186/s12967-021-02996-y. PMID: 34315502 PMC8314639

[B29] Xu T. Yan L. Sun B. Xu Q. Zhang J. Zhu W. . (2022). Impacts of delivery mode and maternal factors on neonatal oral microbiota. Front. Microbiol. 13. doi: 10.3389/fmicb.2022.915423. PMID: 35832807 PMC9271910

[B30] Zemet R. Maktabi M. A. Tinfow A. Giordano J. L. Heisler T. M. Yan Q. . (2025). Amniocentesis in pregnancies at or beyond 24 weeks: an international multicenter study. Am. J. Obstet Gynecol 232, 402.e1–402.e16. doi: 10.1097/01.ogx.0001097904.08725.9a. PMID: 38914189 PMC11663227

[B31] Zhang L. C. Yang X. C. Jiang Y. H. Yang Z. Yan L. L. Zhang Y. X. . (2025). Screening and predictive biomarkers for Down syndrome through amniotic fluid metabolomics. Prenat Diagn 45, 57–69. doi: 10.1002/pd.6693. PMID: 39482571

[B32] Zhu L. Luo F. Hu W. Han Y. Wang Y. Zheng H. . (2018). Bacterial communities in the womb during healthy pregnancy. Front. Microbiol. 9. doi: 10.3389/fmicb.2018.02163. PMID: 30237795 PMC6135892

